# Literary Notices

**Published:** 1876-06

**Authors:** 


### LITERARY NOTICES.

*Scribner's Illustrated Monthly* comes laden with the usual amount of excellent reading. The stories are from the pens of the best writers of fiction. The editor, Dr. Holland, makes a brilliant magazine—one worthy the patronage of professional brethren.

*Lippincott's Illustrated Magazine* stands deservedly high among our literary monthlies. It is copiously illustrated, and well filled with tales of fiction, and other interesting matter.

*Appleton's Journal*, a splendid weekly, is always a source of pleasure and delight. It numbers among its corps ot writers the best talent, among whom are several Southern authors. It will be a welcome visitor in every household.

*St. Nicholas.*—This is the juvenile periodical of the world. It is the prettiest, the best illustrated, the spiciest of all juvenile monthlies. To the young it is "a thing of beauty, and a joy forever." Our friends will do a good thing by placing this delightful little magazine in the hands of their children. Published by Scribner & Co., New York. Price, $3.00.

